# Case Report: Leadless and left bundle branch area pacemakers, complementary advantages require a personalized approach

**DOI:** 10.3389/fcvm.2024.1373884

**Published:** 2024-07-30

**Authors:** Omair Yousuf, Jae (Jeff) Lee, Brett D. Atwater

**Affiliations:** ^1^ Carient Heart & Vascular, Vienna, VA, United States; ^2^ University of Virginia Health, Manassas, VA, United States; ^3^ Inova Schar Heart and Vascular, Inova Health System, Falls Church, VA, United States; ^4^ Virginia Heart, Fairfax, VA, United States

**Keywords:** pacemaker, leadless, conduction system, physiologic pacing, left bundle branch area pacing

## Abstract

Traditional transvenous pacemakers consist of a pacemaker generator usually positioned surgically in the upper left chest on the pectoral muscle fascia and one or more leads positioned through the veins to the right atrium and across the tricuspid valve to the right ventricular apex. While these devices reduce symptoms and improve survival among patients with symptomatic bradycardia, they are associated with an increased risk of infection, venous occlusion, heart failure, and tricuspid valve regurgitation. Although new pacemaker designs minimize these risks, none of the current-generation pacemaker designs effectively eliminate all of them. A personalized approach to selecting the appropriate pacemaker for each patient is needed to optimize outcomes.

## Background

Symptomatic bradycardia can manifest with symptoms of fatigue, shortness of breath, angina, exercise intolerance, pre-syncope, or syncope. Bradycardia can result from disease in the sinus node, called sinus node dysfunction (SND), or from atrioventricular block (AVB). In a pooled analysis of 20,572 patients, whose cases were followed up for 17 years, 291 incident cases of SND occurred, yielding an incidence of 0.8 cases per 1,000 person-years (1). In a large cohort of healthy adults, the prevalence of AVB was 0.1% in people aged <55 years and 0.6% in people aged ≥65 years (2). The pharmacological treatment options for treating SND or AVB are extremely limited, and the most effective treatment strategy includes the implantation of a permanent pacemaker (3). The treatment of SND with permanent pacemaker implantation is associated with improvement in symptoms and a reduction in hospitalizations associated with symptoms of symptomatic bradycardia, such as fatigue, syncope, or exercise intolerance. The treatment of AVB with the implantation of a permanent pacemaker additionally reduces all-cause mortality (3).

The first battery-powered pacemaker surgery was performed in 1958 on a child with AVB after cardiac surgery (4). The device was external and connected to a lead surgically attached to the ventricular epicardium. Subsequently, advancements allowed for the attachment of the lead to the epicardium using a percutaneously inserted needle, avoiding the need for open-heart surgery. However, the pacemaker lead frequently became infected, highlighting the need for a fully implantable pacemaker. The first fully implantable pacemaker surgery was performed on 8 October 1958 at Karolinska Hospital in Stockholm, Sweden (5). Unfortunately, the lead fractured within the first few hours post-implantation, requiring a second surgery for replacement the following morning. Lead infection and failure continue to represent two of the most frequently encountered complications of permanent pacemakers today (6).

### Problems with transvenous pacemakers

**Figure 1:**
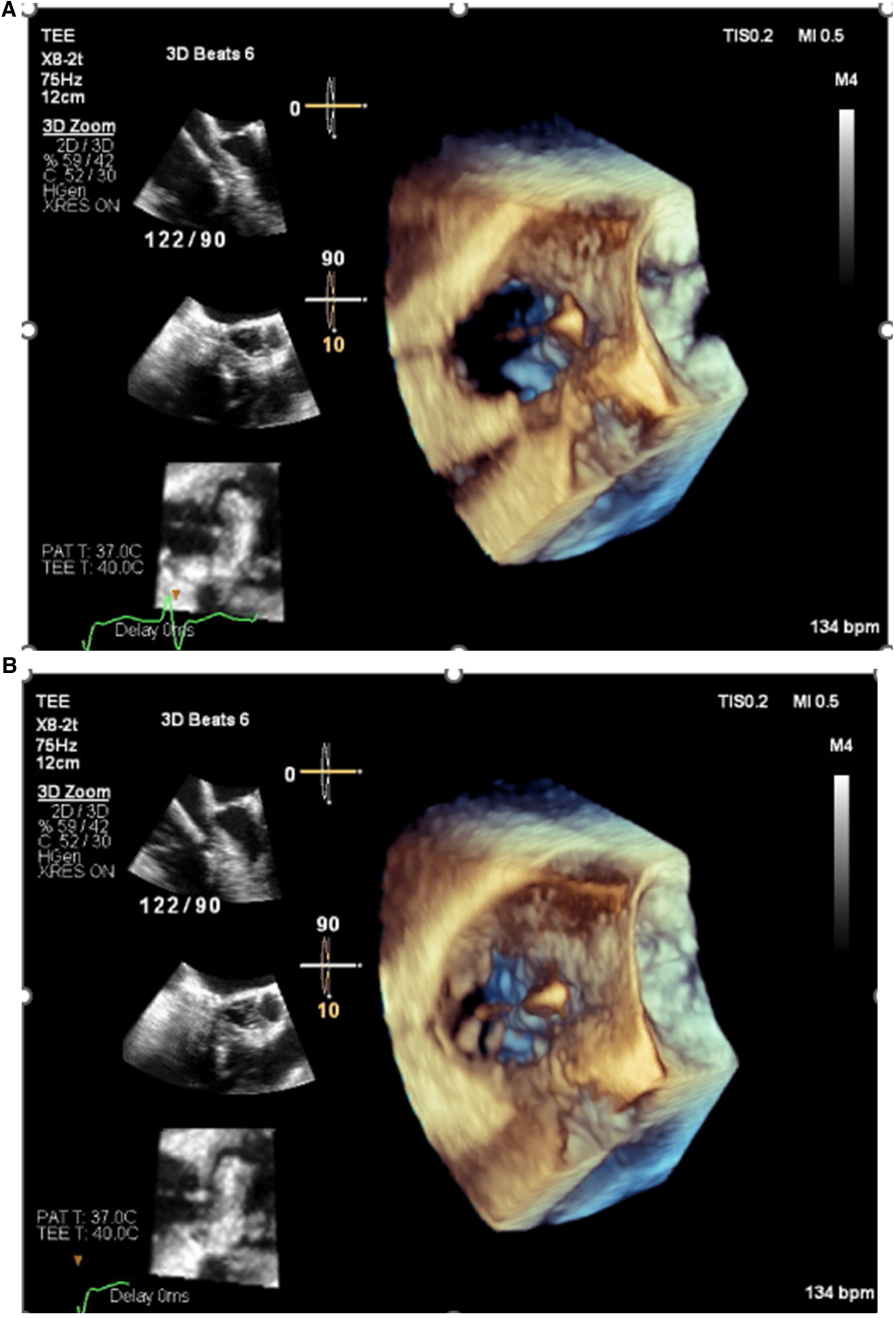
Echocardiogram images demonstrating pinning of the septal leaflet of the tricuspid valve to the septum by a pacemaker lead resulting in severe tricuspid regurgitation. (**A**) Valve open in diastole. (**B**) Valve closed in systole.

**Figure 2:**
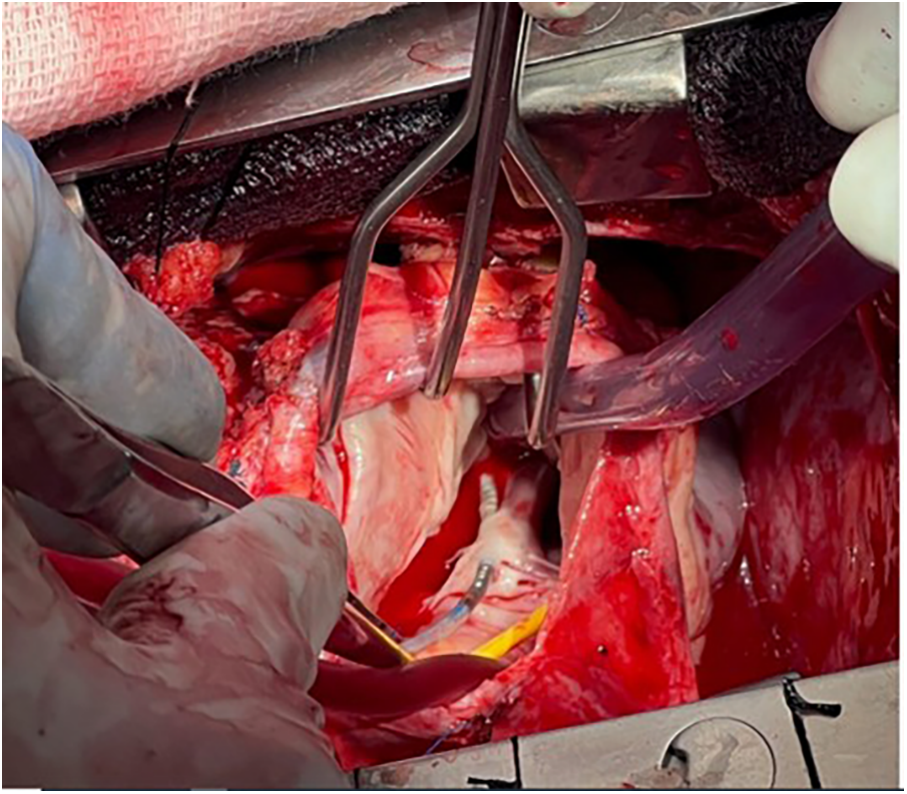
The septal leaflet was punctured by a transvenous pacing lead. This resulted in severe tricuspid regurgitation and heart failure. Surgical repair of the tricuspid valve and repositioning of the lead resulted in a reduction in tricuspid regurgitation and an improvement in heart failure symptoms. (Thanks to Abbas Emaminia, MD, and Eric Sarin, MD, for the photo).

Today, several major problems continue to occur with a relatively high frequency in patients with transvenous permanent pacemakers:

1. Pacemaker leads are subjected to repetitive stress as a result of cardiac, shoulder, and chest motions. After many years, this stress may result in lead failure. In one retrospective study, lead failure occurred in 540/9,782 leads (5.5%) after a mean follow-up of 3.6 ± 2.9 years (7). The risk of lead failure is associated with a variety of factors, including lead design, location of implantation, patient age, and activity level. Lead failure may result in the sudden loss of sensing or pacing and recurrent syncope or death in a pacemaker-dependent patient.
2. Pacemaker leads are routinely placed through a transvenous approach into the heart circulation, making them a source of bloodstream infection, including infective endocarditis. Infection affects 1.2%–2.2% of patients with a traditional transvenous pacemaker (6). Bacteremia leads to the formation of biofilms on infected leads that are resistant to antibiotic treatment alone, necessitating the removal of the entire pacemaker system (8). Lead removal is primarily done through percutaneous extraction, which is a technically challenging procedure associated with significant life-threatening risks (8).
3. Transvenous permanent pacemakers require the surgical formation of a subcutaneous pacemaker pocket, which can confer risks of pocket infection, or pocket hematoma at the site. During surgery, skin flora or bacteria can be introduced into the pocket, which can result in device infection (6, 9). This risk substantially increases with the subsequent generator changes of the pacemaker when the battery is depleted (10).
4. Pacemaker leads are routinely placed in the right ventricle (RV) via the tricuspid valve. Crossing the tricuspid valve can result in valve damage or the development of moderate to severe tricuspid regurgitation. The frequency of significant tricuspid regurgitation after transvenous pacemaker implantation is 10%–20%, ultimately resulting in heart failure symptoms in 50% of those with severe tricuspid regurgitation (11). This can occur as a result of either pinning the valve leaflets against the septum (Figure 1) or accidental puncture of the valve leaflets (Figure 2).
5. Pacing the RV may result in abnormal ventricular electrical activation that leads to left ventricular dyssynchrony, pacing-induced cardiomyopathy (PICM), and subsequent heart failure. Approximately 10% of patients undergoing pacemaker implantation develop PICM with symptomatic heart failure within 2 years of implantation (12). A low RV pacing burden at 20% has been shown to increase the risk of heart failure, mortality, and hospitalization compared to normal conduction. Guidelines recommend the use of pacing algorithms that minimize ventricular pacing and are primarily used in patients with SND, but their applicability is limited in patients with AVB (13).
6. Transvenous pacing leads may cause occlusion of the axillary, innominate, or superior vena cava resulting in superior vena cava syndrome, swelling of the ipsilateral arm, and patient discomfort. Venous stenosis or occlusion is observed in 20%–60% of patients with transvenous devices and is associated with the number of leads, number of lead implant procedures performed, and total lead diameter (14).
7. Device migration and chronic patient discomfort affect the device performance and quality of life after transvenous pacemaker placement. In some cases, device migration or chronic pain may necessitate repeated procedures to revise the pacing system. In one study of 16,517 patients undergoing cardiac electronic implantable device procedures at the Mayo Clinic, 20.8% of patients required opioid pain medications after the procedure, while 1.5% required new chronic opioid use due to chronic discomfort (15). Device or lead migration occurs most commonly in children, obese patients, patients who are very physically active, and those with “Twiddler’s syndrome.”

Recent pacemaker research has focused on reducing the frequency of these problems through the development of leadless and conduction system pacing via His bundle or left bundle branch area pacemakers (LBBAP). This review focuses on the advantages and disadvantages of these solutions and the future of cardiac pacemakers.

## Leadless pacemakers: eliminate the lead, eliminate the problems?

The basic design of transvenous cardiac pacing devices remained unchanged from the mid-1960s until the early 2010s (16). Clinicians and scientists focused on reducing pacemaker-related infectious complications through improvements in surgical techniques, perioperative antibiotic selection, and incorporation of antimicrobial-coated envelopes around the device (17), while improvement in lead design helped reduce lead failures. Despite improvements in design and implantation techniques, lead fracture and device-related infections remained as the frequent causes of pacemaker-related complications. To address this, leadless pacemakers were developed and first became commercially available in 2016. Leadless pacemakers are completely self-contained implantable devices capable of pacing, sensing, and communicating wirelessly. They are implanted via the femoral or internal jugular vein using a catheter delivery system and deployed in the RV septum or apex.

By eliminating the pacemaker lead and pocket, the leadless pacemaker implantation procedure is simplified and the frequency of long-term complications is reduced by almost 50% compared to transvenous pacemakers (18). Lead fracture and venous occlusion are completely eliminated, the risk of damage or impairment of the tricuspid valve is minimized, and no cases of leadless pacemaker-associated infection have been reported in clinical trials enrolling more than 3,000 patients (19). More than 150,000 leadless pacemakers have been implanted worldwide, with only four reported cases of device infection requiring removal (20). Initially, leadless pacemakers were single-chamber devices capable of pacing and sensing the RV. Subsequent improvements led to the ability to sense atrial activity and provide reasonable tracking with AV synchrony in patients with AVB. In July 2023, the FDA approved the first dual-chamber leadless devices, which consist of two devices, one implanted in the right atrium and one in the RV, making leadless pacing a treatment option for a wide range of indications, including those with SND (21).

### Problems with leadless pacemakers

1. Unfortunately, current-generation leadless pacemakers cannot pace the left ventricle or conduction system. As a result, patients remain at risk for PICM. Ventricular dyssynchrony has been shown to cause a 10% reduction in the LV ejection fraction (EF) in the first 7 days of pacing and results in heart failure in 10% of patients (12). It is unclear whether PICM and heart failure occur at the same frequency in patients with leadless vs. transvenous devices. One single-center study showed that the frequency may be lower in patients with leadless pacemakers, while others showed a similar incidence of PICM after leadless and traditional transvenous RV pacemakers (22, 23). Finally, the position of the leadless pacemaker within the RV may predict the incidence of PICM after implantation. In a retrospective study of 358 patients, PICM occurred in 4% of patients with a high or mid-RV septal position and in 16.5% of patients with an apical septal location (24).
2. Leadless pacemaker generators may be more difficult to remove and replace than traditional pacemaker generators. The current battery longevity estimates for single-chamber leadless pacemakers that are commercially available in the USA and Europe range from 5 to 20 years depending on percentage pacing, capture thresholds, and single or dual design. Younger patients who require extended pacemaker performance may need multiple reimplants as devices reach the end of service. It remains unclear whether removing the original implant at the end of service improves or worsens patient outcomes.
3. Ventricular leadless pacemakers that do not track the atrium (VVI or VDD with inadequate atrial sensing) may cause pacemaker syndrome. Pacemaker syndrome occurs in up to 20% of patients in sinus rhythm who are paced in VVI mode and is characterized by lightheadedness, shortness of breath, fatigue, and heart failure symptoms (25). Pacemaker syndrome is best treated with the restoration of atrioventricular synchrony through device reprogramming or implantation of a dual-chamber device.

## Conduction system pacemakers: preventing and treating pacemaker-induced and left bundle branch block-mediated cardiomyopathies

While leadless pacing has addressed the long-standing problems of lead fracture, venous occlusion, and device infection, conduction system pacing addresses the problems of dyssynchrony and PICM. Conduction system pacing is performed by pacing either the His bundle or the left bundle branch. Due to superior short- and long-term performance, left bundle branch area pacing (LBBAP) now comprises >90% of implanted conduction system pacemakers. LBBAP can be performed by accessing the left ventricular conduction system by implanting the pacemaker lead from the RV through the interventricular septum. In contrast to RV pacing, PICM has not been reported after successful LBBAP implantation. The implantation time and complication frequency are similar to RV lead placement (26) after sufficient experience. The most frequent complications are lead dislodgement and perforation of the ventricular septum into the LV (26). In addition to its usefulness in the setting of AV block, LBBAP may also be a suitable alternative to biventricular pacing (BIVp) for patients with underlying dyssynchrony occurring in the setting of a wide intrinsic QRS, particularly from left bundle branch block (27). The implantation success defined using ECG criteria to verify left bundle branch capture is 92% for patients without heart failure and 82% for patients with heart failure (26). LBBAP for delivery of cardiac resynchronization therapy (CRT) may reduce lead burden and potentially reduce lead-related complications, including venous stenosis and infection, compared to BIVp as resynchronization can be performed via one rather than two ventricular leads.

A recent prospective study comparing LBBAP and BIVp as an initial strategy in those referred for CRT demonstrated lower rates of the composite endpoint of heart failure hospitalization and mortality for patients treated with LBBAP compared to those treated with BIVp (28). A large prospective randomized controlled trial comparing LBBAP and BiVp for treatment of HF in the setting of LBBB or high expected pacing burden is currently enrolling (NCT05650658).

Because LBBAP is relatively new, limited data are available regarding the long-term durability or ease of extraction of leads placed in the left bundle area. A series of case reports, consisting of <10 patients who underwent extraction of both lumenless and stylet-driven leads, show that the majority of leads can be successfully removed using manual traction and counterclockwise rotation of the lead. One report using mechanical extraction tools demonstrated that the distal lead helix was retained in the septum after extraction, but this did not result in an adverse patient outcome (29).

## A difficult decision: transvenous RV pacing, transvenous left bundle branch area pacing, or RV leadless pacing?

**Figure 3:**
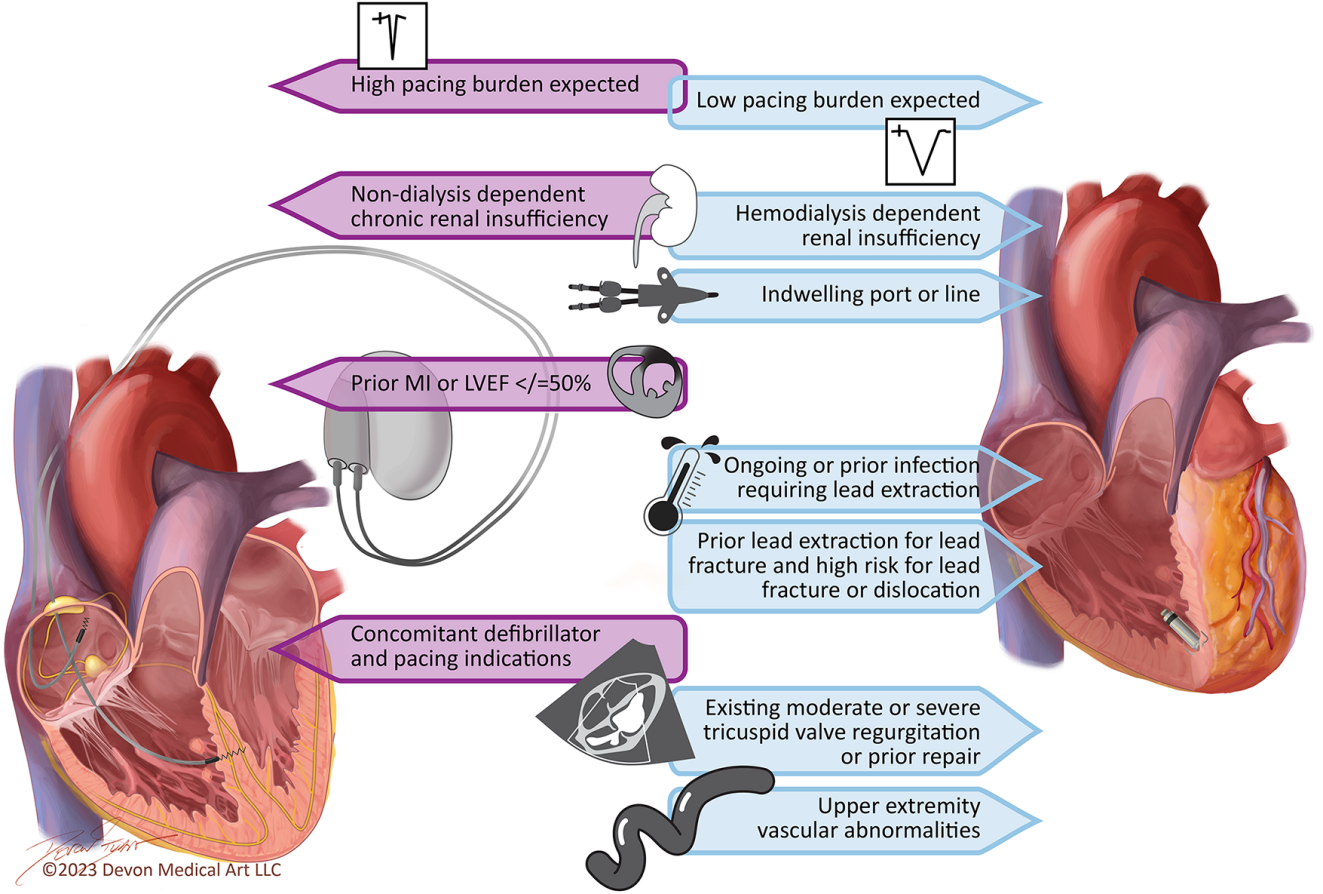
Key considerations for the pacemaker selection in patients with ejection fraction >35%. Patients with an ejection fraction of 36%–50% and a high expected pacing burden or left bundle branch block with heart failure symptoms may also qualify for biventricular pacing.

In an ideal world, a pacing system could mitigate the challenges associated with transvenous leads and allow for conduction system pacing. A leadless LBBAP system could provide ventricular stimulation with a lower risk of lead fracture, infection, damage to the tricuspid valve, and PICM compared to current-generation devices. Such devices are currently in development, but the design of these systems has been proven as complex. For the time being, implanters must make a decision: choose a transvenous RV pacemaker that may cause PICM, tricuspid valve damage, vascular occlusion, and infection, choose an LBBAP pacemaker that may reduce the risk of PICM but retains the risks of lead- and pocket-related complications, or choose a leadless pacemaker without lead- and pocket-related complications that retains the risk of PICM and may increase the risk of pacemaker syndrome. The decision must be personalized to the individual needs of the patient being treated. Figure 3 presents the clinical characteristics that should be considered when choosing between leadless RV pacemakers and transvenous LBBAP pacemakers in patients with an EF of >35%.

Patients who have an indication for an implantable cardioverter–defibrillator in addition to the need for pacing currently require a transvenous device with an RV lead location. Many of these patients have a depressed EF and require a left ventricular or LBBAP lead and a biventricular defibrillator generator to reduce the probability of PICM. Ongoing studies are investigating the safety of defibrillator leads capable of pacing the left bundle area and a system employing a leadless pacemaker and a subcutaneous defibrillator that are capable of communication (30). Future studies are needed to determine if these systems can reduce the probability of device-related adverse events in patients who have indications for both defibrillator and pacing therapy.

The 2023 HRS/APHRS/LAHRS Guideline on Cardiac Physiologic Pacing for the Avoidance and Mitigation of Heart Failure suggests a Class 2b indication for conduction system pacing for patients with a left ventricular EF of >50% who are expected to require >20%–40% ventricular pacing and a Class 2a indication for conduction system pacing or BIVp in patients with an EF of 35%–50% who are expected to have >20%–40% ventricular pacing (31). We currently have no prospective data comparing the outcomes for transvenous RV pacing and LBBAP in patients with an EF of >50% and an expected ventricular pacing burden of <20%, and there are no guideline recommendations for the use of LBBAP in this patient group. However, it may be difficult to accurately predict which patients will require >20% ventricular pacing at the time of device implantation when the decision between devices needs to be made. Patients with complete or high-grade AV block may be expected to have a ventricular pacing burden of >20%, but 10%–20% of patients with a SND indication for pacemaker implantation may end up requiring >20% ventricular pacing at 3- and 6-year follow-up. Moreover, the positive and negative predictive values of the operator opinion for accurately predicting ventricular pacing burden are lower in patients with SND (87% and 88%, respectively) compared to those with AV block (92% and 100%, respectively) (32).

Patients with preexisting LV dysfunction or with characteristics associated with a high probability of developing PICM, including those expected to have a high burden of ventricular pacing, prior MI, renal insufficiency not requiring dialysis, or male sex (12), may benefit from preferential implantation of a transvenous LBBAP over transvenous or leadless RV pacemakers. The 2021 EHRA/HRS/LAHRS/APHRS position paper on the use of leadless pacemakers highlights that patients with two or more risk factors for infection (e.g., prior device infection, chronic immunosuppressive therapy, ongoing bloodstream infection or fever, poorly controlled diabetes mellitus, chronic indwelling catheter or port, and ongoing or expected hemodialysis) may benefit from a leadless pacemaker (33). It is important to highlight that the 4%–16% incidence of PICM observed among patients with leadless pacemakers (22–24) exceeds the reported risk of device-related infection (1%–2%) and other lead-related complications (2.8%) in patients with LBBAP (26). Therefore, in our practice, patients without contraindications to a transvenous system, who are expected to require a high burden of ventricular pacing, preferentially receive LBBAP over leadless pacemakers to reduce overall pacemaker-related complication rates. Further prospective trials investigating the relative risks of transvenous LBBAP and leadless RV pacemakers in this population are needed.

Cases 1 and 2 below highlight the potential advantages of each of these pacing therapies in appropriately selected patients. Shared decision-making between physicians and patients should be used to help determine optimal device selection.

### Case 1

**Figure 4:**
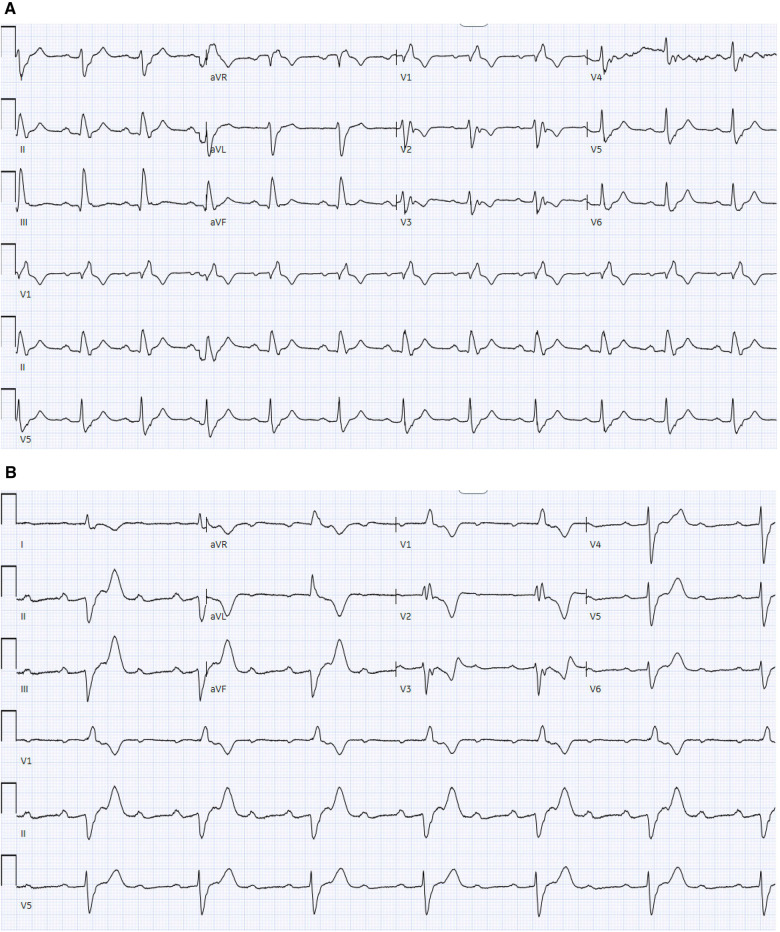
(**A**) 12-lead ECG demonstrates sinus rhythm with RBBB without AV block. (**B**) 12-lead ECG demonstrates sinus rhythm with a 2:1 atrioventricular block with RBBB and prolonged AV conduction.

**Figure 5:**
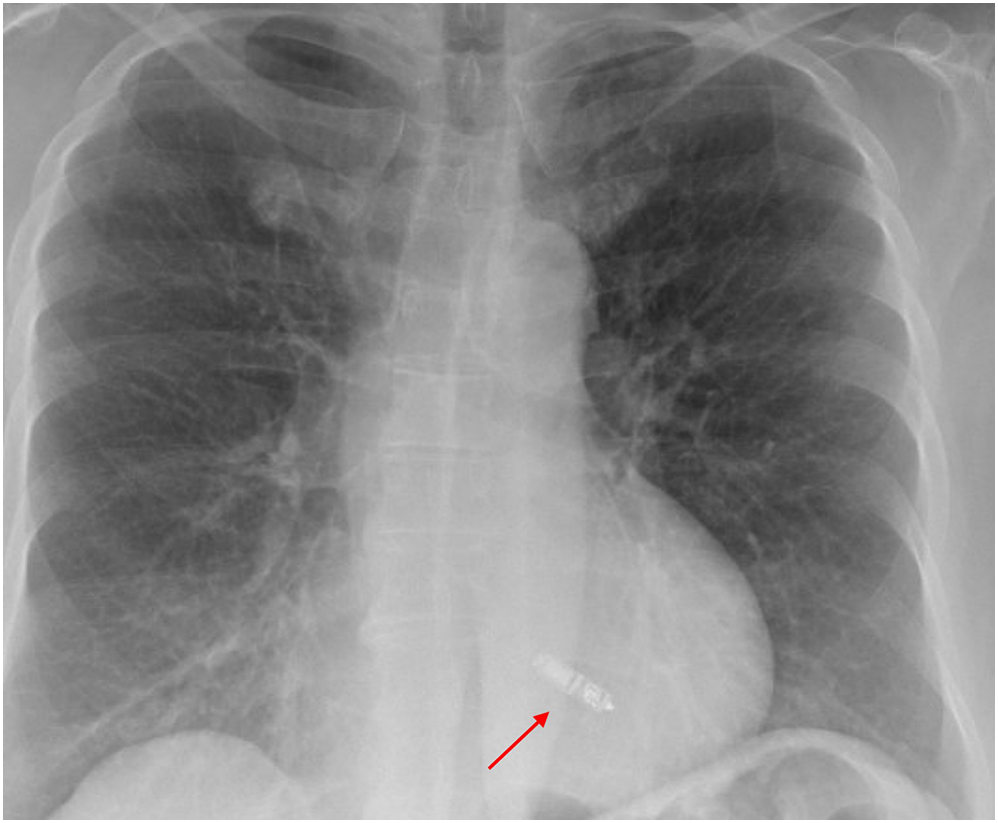
Chest x-ray demonstrating a Medtronic Micra leadless pacemaker implant (red arrow).

A 72-year-old woman with a history of hypertension, diabetes, and coronary artery disease with prior percutaneous coronary intervention to the left anterior descending artery presented with fatigue and intermittent lightheadedness. She had no history of syncope. Her vital signs were unremarkable. Laboratory evaluation, including metabolic profile, thyroid testing, and blood counts, was also unremarkable. The echocardiogram showed an EF of 60%–65%, mild mitral regurgitation, and mild concentric left ventricular hypertrophy. The ECG results showed sinus rhythm with right bundle branch block (Figure 4A). She was monitored on telemetry and was noted to have paroxysmal 2:1 AV block with ventricular rates of 30–35 bpm (Figure 4B) associated with pre-syncope. She underwent a transvenous dual-chamber LBBAP implant for symptomatic bradycardia. The patient presented 2 weeks later for a routine office visit and was noted to have erythema around the incision site with a small superficial dehiscence noted on the medial edge of the incision. She had no fever. Her laboratory evaluation was notable for a normal white blood cell count, and the blood cultures remained negative. She was prescribed 7 days of cephalexin 500 mg QID. Unfortunately, she presented again 10 days later with wound dehiscence and purulent drainage. She was admitted for pacemaker explant. The leads were easily removed using manual traction without the need for extraction tools. The pocket cultures grew *Staphylococcus epidermidis.* The blood cultures and lead tip cultures remained negative. She was treated with daptomycin for 2 weeks. After careful consideration of the risks and benefits of reimplanting an LBBAP from the right chest or a leadless AV pacemaker, she underwent implantation of a leadless AV pacemaker, as shown in the chest x-ray taken the following morning (Figure 5).

A pacemaker-related infection is associated with high morbidity and mortality and contributes to high healthcare-associated costs and utilization. Infections may be limited to the pacemaker pocket site or may become systemic as a result of bacterial seeding of the hardware or intravascular spread from an infected pocket. In systemic infections, mortality may be as high as 25%, and incremental healthcare costs can exceed $16,000/case in the USA (34, 35).

In the pivotal trial and post-approval studies of over 3,000 Medtronic Micra leadless pacemaker implants, no device-related infections were reported (36, 37). In the investigational device exemption study, among 720 leadless implants, 16 patients developed bacteremia or endocarditis. The most common organism was *Staphylococcus aureus*. All patients responded to appropriate antibiotic therapy, and none required extraction (37).

The most significant advantage of the leadless pacemaker compared to transvenous LBBAP is the absence of a subcutaneous pocket and transvenous leads, which significantly reduces the hardware burden and eliminates any direct communication with the skin. The rate of infection in transvenous implants may be as high as 2.3% (38, 39), with 60% presenting with pocket infection and 40% with bacteremia (39). Endothelialization and encapsulation of the leadless pacemaker within the RV may contribute to a lower risk of infections.

In this case, we elected to reduce the risk of recurrent pacemaker-related infection as much as possible while accepting the risk of PICM by choosing to implant a leadless pacemaker. This difficult decision was made using a shared decision-making process that involved the patient and her family in a thorough discussion.

### Case 2

**Figure 6:**
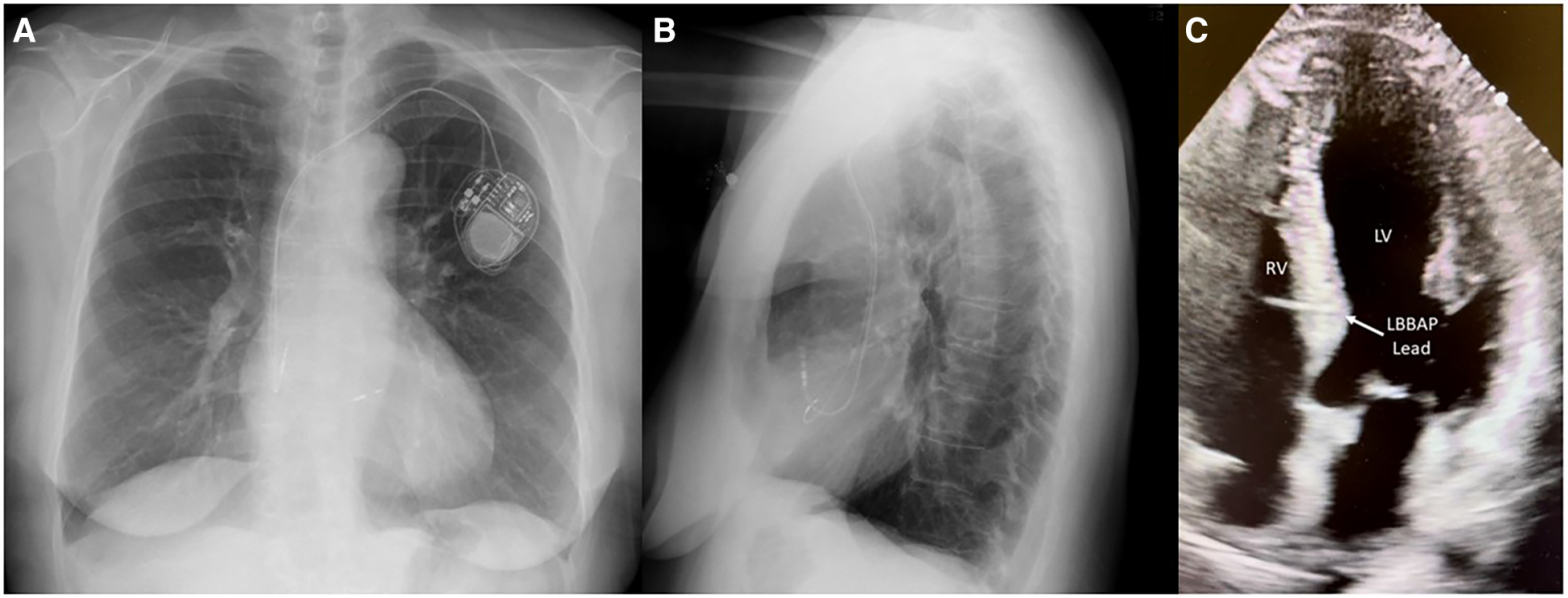
Medtronic 3830 lead with a fixed helix implanted through the RV endocardium to the LV endocardium in the mid-interventricular septum capturing the left bundle branch of the conduction system. (**A**) PA chest x-ray, (**B**) lateral chest x-ray, and (**C**) four-chamber transthoracic echocardiogram view of the left bundle branch pacing lead.

**Figure 7:**
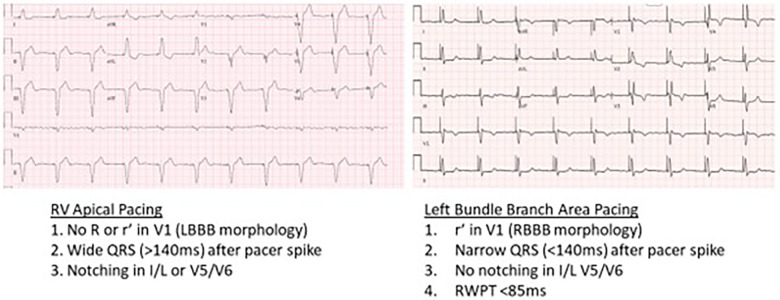
12-lead ECGs demonstrating RV apical pacing vs. LBBAP.

A 68-year-old man with a history of hypertension presented with two recent episodes of syncope. He denied any recent travel or tick exposure. His vital signs were notable for a heart rate of 35 bpm and a blood pressure of 164/88. His laboratory evaluation, including metabolic profile, blood counts, troponin, and thyroid levels, was unremarkable. The 12-lead electrocardiogram revealed sinus rhythm with a third-degree AVB and escape rhythm with a ventricular rate of 30 bpm. The echocardiogram revealed an EF of 50% with trace tricuspid regurgitation. A temporary transvenous pacemaker was placed urgently via the right internal jugular vein, and an LBBAP was implanted the following day. The chest x-ray (Figures 6A,B) and echocardiogram (Figure 6C) results demonstrated a Medtronic 3830 lead with a fixed helix implanted through the RV endocardium to the LV endocardium in the mid-interventricular septum, capturing the left bundle branch of the conduction system. A post-implant ECG demonstrated atrial and ventricular paced rhythm. The ventricular paced morphology was consistent with the left bundle branch capture, as demonstrated by a prominent terminal *r*′ in lead V1 (Figure 7) and a V6 R wave peak time of 68 ms and as measured from the pacing stimulus to the peak of the R wave in lead V6. The paced QRS duration was 112 ms.

Current guideline recommendations support CRT with BIVp in patients with symptomatic heart failure, a left ventricular EF of ≤35%, and an anticipated ventricular pacing burden of >20% or a preexisting conduction block with a QRS duration >120 ms. Conduction system pacing using His or LBBAP approaches received a Class 2a indication for patients with an EF of 36%–50% and an expectation of a high ventricular pacing burden (31) However, as many as 30% of patients do not respond to CRT delivered by BIVp. LBBAP has been shown to reduce mortality, heart failure hospitalization, and the need for an upgrade to BIVp as compared to RV pacing (40–42).

In this case, we elected to reduce the risk of PICM as much as possible, while accepting the potential for lead-related complications. This decision was made after considering the expected high burden of ventricular pacing and the baseline mildly depressed EF. The patient had none of the high-risk characteristics associated with infection.

## Conclusion

Leadless and LBBAP implantations address the most common problems encountered after traditional transvenous RV pacemaker implantation. Accurately predicting the risks for PICM, infection, lead failure, and venous occlusion and personalizing the decision between leadless and LBBAP while using a shared decision-making process will provide optimal patient outcomes while reducing healthcare costs and procedure-related complications.
